# Research on sampling of COVID-19 with lidocaine-soaked swab

**DOI:** 10.1097/MD.0000000000043918

**Published:** 2025-08-15

**Authors:** Chen Gong, Xiaoyan Lan

**Affiliations:** a Respiratory Medicine Department, The First Affiliated Hospital of Guangxi Medical University, Nanning, Guangxi Zhuang Autonomous Region, People’s Republic of China; b Experiment Center, Faculty of Chinese Medicine Science Guangxi University of Chinese Medicine, Nanning, Guangxi Zhuang Autonomous Region, People’s Republic of China.

**Keywords:** COVID-19, Lidocaine, nasopharyngeal swab, oropharyngeal swab, SARS-CoV-2

## Abstract

**Background:**

This study explores the comfort level of lidocaine-soaked swab for nucleic acid detection of COVID-19 (Coronavirus Disease 2019) patients. Oropharyngeal and nasopharyngeal secretions from COVID-19 patients were collected using lidocaine-soaked swabs.

**Methods:**

COVID-19 patients were randomly divided into experimental group and control group, with 25 patients in each group. The first oropharyngeal and nasopharyngeal swabs (experimental group) was be soaked in lidocaine hydrochloride injection. The control group was tested for novel coronavirus nucleic acid by routine nasopharyngeal/oropharyngeal swabs. Then, severe acute respiratory syndrome coronavirus 2 (SARS-CoV-2) ribonucleic acid (RNA) was detected in 2 groups by real-time reverse transcription-polymerase chain reaction (RT-PCR). Participants’ discomfort was assessed by a visual analog scale. Then, the discomfort and cycle threshold (Ct) values after sampling were compared between the 2 groups.

**Results:**

A total of 50 patients were enrolled in the study. There were no lidocaine-related complications during sampling. COVID-19 inpatients feel more comfortable with the use of lidocaine-soaked swabs (nasopharyngeal 3.44 ± 1.42 vs 5.68 ± 0.95, *P* < .01; oropharyngeal 4.4 ± 1.53 vs 8.92 ± 0.91, *P* < .01). Ct values of nasopharyngeal swab specimens were generally lower than those of oropharyngeal swab specimens. There was no statistically significant difference between Ct value results of the 2 groups. The use of lidocaine-soaked swabs for sampling of COVID-19 reduces patients’ discomfort. This method is worth popularizing in novel coronavirus sampling.

**Conclusion:**

The use of lidocaine-soaked swabs for sampling of COVID-19 reduces patients’ discomfort, which it has no obvious effect on Ct value. This method is worth popularizing in novel coronavirus sampling.

## 1. Introduction

SARS-CoV-2 began to outbreak at the end of 2019, so far, it has spread to the world. Currently, Omicron strains infection has replaced Delta strains as major epidemic strains. Existing evidence suggests that the propagation of Omicron force stronger than Delta, pathogenicity weakened.^[1]^ Regular use of polymerase chain reaction detection diagnosis accuracy is not affected. Most patients are asymptomatic or light. So many patients unable to detect their infected COVID-19. So, regular SARS-CoV-2 nucleic acid testing is necessary, especially in frontier ports, hospitals, cold chain food transportation, and other high-risk occupational exposure workers.^[2]^

The present study showed that results of nasopharyngeal swab sampling have a higher positive rate of SARS-CoV-2 nucleic acid than oropharyngeal swab.^[3]^ It reduces missed diagnosis and minimizes false negative rate. Due to the deep insertion and friction on the wall of the nasopharyngeal cavity during nasopharyngeal swab sampling, the subjects showed pain, sneezing, nausea, cough, and a few showed tears and other discomfort,^[4]^ especially in the relatively difficult collection group, such as infants and children. Therefore, the vast majority of subjects refused to take nasopharyngeal specimens. However, sporadic cases exist in Chinese many places. There is an increasing trend of outbreaks in some areas (Shanghai, Jilin, Baise, China). It is of great significance to take nasopharyngeal specimens for novel coronavirus nucleic acid detection.

In this study, the specimen were collected using lidocaine-soaked disposable virus swabs. Compared with previous standard collection methods, this method whether there is any difference in pain score and virus Ct detection value, explores a new method of collecting novel coronavirus nucleic acid, alleviates the pain of subjects, and also provides a partial reference for policy promotion.

## 2. Methods

The study population included patients admitted to the isolation wards of Chongzuo Hospital from March 2022 to May 2022. The inclusion criteria were patients aged between 18 and 65 years old who had been diagnosed with COVID-19 and required inpatient treatment. The exclusion criteria were patients younger than 18 or older than 65, pregnant or lactating women, patients with allergies to lidocaine, and patients with severe underlying diseases or critical illness in the ICU (Intensive Care Unit), as defined by the “Diagnosis and Treatment Guidelines for COVID-19 (Trial Version 9).” Initially, 56 patients were included, but due to concerns about the experimental study and lack of time, 50 patients were finally included. The enrolled patients were randomly divided into an experimental group and a control group using a random number table, with 25 patients in each group. The study was approved by The First Affiliated Hospital of Guangxi Medical University and Chongzuo Second People’s Hospital ethics committee and complies with the Declaration of Helsinki of the World Medical Association. All patients signed informed consent.

COVID-19 patients were randomly divided into experimental group and control group, with 25 patients in each group. Samples from the control group were collected with standard disposable swabs. Samples from experimental group were collected with swabs which was be soaked in lidocaine hydrochloride injection (5ml:0.1g, Shanghai Harvest Pharmaceutical Co., LTD). All specimens were collected by the same professionally trained nurse. The operation process of swab technology includes the following steps: check the information of the examinee and explain it appropriately to eliminate their nervousness. Check the nasal cavity, instruct the patient to remove the mask, tilt the head back, and use a flashlight to check for abnormalities such as nasal mucous membrane bleeding and nasal septum curvature.

Before opening the swab bag, measure the distance from the nostril to the earlobe with a swab and mark the corresponding position on the swab handle. For sampling, gently hold the head of the examinee with 1 hand, insert the swab into the nostril in a vertical (face) direction with the handle of the swab marked position reaching the nostril position, gently rotate it for 1 circle; then slowly remove the swab, dip the swab head into a tube containing 2 to 3 mL virus preservation solution; finally remove the swab tail along the crease and tighten the cap. Send for inspection, recheck the information of the examinee, and send for inspection in a timely manner after verification. After each collection, the sampling personnel should perform hand disinfection. The specimens (2 groups) were placed in disposable virus sampling tube (Rich Science, Chendu, China) and transported in novel coronavirus nucleic acid testing laboratory, including ORF (Open Reading Frame) 1ab gene, N (Nucleocapsidencoding) gene of SARS-CoV-2.

Participants’ discomfort was assessed by a visual analog scale (VAS) and Patient symptom questionnaire. VAS is a pain assessment tool that uses a 10-cm straight line with 2 ends representing no pain (0 point) and the most severe pain (10 points). The patient marks the pain level on the line based on their own experience. This method is simple and widely used in clinical practice. After sampling specimen, the patient marked a point corresponding to the pain intensity on a straight line with a pen according to their own situation. Pain was measured on a scale of 0 to 10. Repeat 2 times and take the average of 2 times. Half an hour after sampling, patients should be observed for lidocaine-related complications. The patient will be given a symptom questionnaire survey 6 to 8 hours after completing the throat swab. The patient symptom questionnaire included symptoms such as fever, cough, sore throat, nasal obstruction, sneezing, and diarrhea to investigate the symptoms of patients after completing throat swabs.

This study used the statistical software SPSS 26.0 (Chicago) for data analysis. Count data can be described by the number of cases, and comparisons were made using x^2^ tests. Measurement data were subjected to K–S normality testing, and those that met normal distribution were described using mean ± standard deviation (x ± s), and comparisons were made using *t*-tests. Those that did not meet normal distribution were described using median (lower quartile, upper quartile) (M [Q1, Q3]), and comparisons were made using nonparametric tests, comparisons were made using rank sum tests. Statistical analysis Ct values of the RT-PCR of the 2 groups with lidocaine (experimental group) and without lidocaine (control group) were compared for any statistical significance using *t*-test for 2 independent samples. The *t*-test of 2 independent samples was also used to compare the discomfort scores on VAS. *P* < .05 was considered significant.

## 3. Results

Fifty adult patients were included in the study. Clinical characteristics profile is shown in Table 1. There were 21 asymptomatic infected patients, 29 cases of mild COVID-19, no severe patients. Among them, the main clinical manifestations of mild patients are fever, cough, Sore throat, nasal obstruction and diarrhea.

**Table 1:** Clinical characteristics of patients between the 2 groups.

Clinical characteristics	Experimental group	Control group	*t*/χ2	*P*
Age (year)	31.01 + 24.74	25.99 + 22.52	1.198	.237
Gender				
Male	11	7	1.389	.239
Female	14	18		
Clinical symptoms				
Fever	2	3	0.222	.637
Cough	4	3	0.166	.684
Sore throat	6	4	0.500	.48
Nasal obstruction	2	1	0.355	.552
Diarrhea	1	3	1.087	.297
Clinical classification				
Asymptomatic	10	11	0.082	.774
Light	15	14		

COVID-19 patients of experimental group feel more comfortable by lidocaine-soaked swabs than patients of control group (Table 2). VAS scores for discomfort were 3.44 ± 1.42 by sampling oropharyngeal specimens in experimental group. Nevertheless, those scores were 5.68 ± 0.95 without lidocaine group (control group). The difference was statistically significant on VAS discomfort scores between the 2 groups. (*P* < .01). Nasopharyngeal sampling is often more uncomfortable than oropharyngeal sampling. VAS scores were 4.4 ± 1.53 for COVID-19 patients with lidocaine-soaked swabs in lidocaine by sampling nasopharyngeal specimens, while they were 8.92 ± 0.91 for the control group. The experimental group was significantly less uncomfortable than the control group (*P* < .01).

**Table 2:** Visual analog scale scores between the 2 groups (Mean ± SD).

Swab	Experimental group	Control group	*t*	*P*
Oropharyngeal swab	3.44 ± 1.42	5.68 ± 0.95	−6.577	<.001
Nasopharyngeal swab	4.4 ± 1.53	8.92 ± 0.91	−12.713	<.001

SD = standard deviation.

According to Ct value results of oropharyngeal swab specimens, Ct value of ORF 1ab gene for the experimental group was 34.59 ± 4.22 while it was 36.01 ± 3.75 for the control group (*P* = .215), Ct value of N gene for the experimental group was 34.02 ± 4.22 while it was 34.34 ± 4.62 for the control group (*P* = .800). According to Ct value results of nasopharyngeal swab specimens, Ct value of ORF 1ab gene for the experimental group was 27.60 ± 5.52 while it was 28.79 ± 4.01 for the control group (*P* = .385), Ct value of N gene for the experimental group was 26.80 ± 5.44 while it was 28.70 ± 3.04 for the control group (*P* = .135). The difference was on statistically significant between the 2 groups (Table 3).

**Table 3:** The Ct values of the ORF 1ab gene and N gene between the 2 groups (Mean ± SD).

Gene	Experimental group	Control group	*t*	*P*
Oropharyngeal swab				
ORF 1ab gene	34.59 ± 4.22	36.01 ± 3.75	−1.256	.215
N gene	34.02 ± 4.22	34.34 ± 4.62	−0.255	.800
Nasopharyngeal swab				
ORF 1ab gene	27.60 ± 5.52	28.79 ± 4.01	−0.877	.385
N gene	26.80 ± 5.44	28.70 ± 3.04	−1.519	.135

Ct = cycle threshold, ORF = open reading frame, SD = standard deviation.

## 4. Discussion

At present, novel coronavirus nucleic acid samples of patients are collected through upper and lower respiratory tract. Upper respiratory tract specimens are easy to collect and are suitable for patients with asymptomatic and mild symptoms. Meanwhile, the novel coronavirus nucleic acid test results of nasopharyngeal swabs in upper respiratory tract specimens are better than those of pharyngeal swabs.^[5]^ In this study, Ct values of nasopharyngeal swabs of novel coronavirus infected patients were significantly lower than those of pharyngeal swabs. It is more reliable to measure nucleic acid by nasopharyngeal specimens. The probable cause is the peculiar anatomy of the nasal cavity. SARS-CoV-2 is more likely to hide in the nasal mucosa, and the mucus blanket of the nasal mucosa may have adsorption effect on SARS-CoV 2. Nasopharynx is the first portal for the virus to invade the human body. Virus infection generally originates in the upper respiratory tract, develops from nasopharynx to pharynx, and finally attacks the lower respiratory tract.

Pain is a subjective feeling of patients, so there is no objective medical instrument for the evaluation of pain intensity, which mainly depends on the subjective description of patients. In this study, the use of lidocaine-soaked swabs for nasopharyngeal and oropharyngeal specimens significantly reduced patients’ discomfort. The study of Kanodia^[6]^ was the first to find that external use of lidocaine reduced patient discomfort without affecting sample quality during SARS-CoV-2 sample collection. However, his study told patients to suck lidocaine lozenges, which can only be used in the oropharynx, and are time-consuming and costly. In this study, modified swab samples soaked with lidocaine were applied to both nasopharynx and oropharynx. Wilson et al reported that coughs and sneezes, which result in large amounts of virus excretion, should be minimized^.[7]^ Virus particles may hang in the air for some time, which will make the area difficult to disinfect, leading to a higher risk of transmission. Therefore, minimizing upper respiratory tract reflexes is necessary and can be achieved with a soaked lidocaine swab.

In the design of our study, we also considered other ways of giving lidocaine in the form of aerosols and topical sprays, which are not suitable for use in COVID-19 patients because they produce aerosols.^[8]^ In our study, lidocaine-soaked swabs showed no special discomfort or lidocaine-related allergic reactions, except for a few patients who felt slightly bitter during sample collection. Will the sample collection method with lidocaine intervention affect the test result of Ct value? In our study, it was found that there was no significant difference in Ct values between 2 groups. Therefore, swabs soaked with lidocaine did not affect the nucleic acid test results of COVID-19.

As a local anesthetic, lidocaine can alleviate the discomfort during throat swab collection, thereby improving patient comfort and compliance. Lidocaine can also inhibit the inflammatory response of the respiratory mucosa, reducing symptoms such as coughing and runny nose that may occur during the collection process, making the collection process smoother. By comparing the Ct values and VAS scores of the 2 groups, as well as symptom surveys, we can more accurately evaluate the effect of lidocaine during throat swab collection, providing a reference for subsequent clinical practice. At the same time, this study also has limitations, with a relatively small sample size that may not fully represent the actual situation of different age, gender, and disease condition. It needs to be verified in larger-scale studies. At the same time, the use of lidocaine may cause adverse reactions in some patients, such as skin itching, and arrhythmia, which need to be closely observed and taken corresponding measures in clinical practice. Since this method has not been widely used and promoted, large-scale patients have not been included. Meanwhile, the number of patients in this region is limited, and a third placebo group has not been established. In the future, we will apply this study to routine screening of high-risk population to further expand the sample size and improve the persuasiveness of this method.

## Author contributions

**Data curation:** Xiaoyan Lan.

**Writing – original draft:** Chen Gong, Xiaoyan Lan.

**Writing – review & editing:** Chen Gong, Xiaoyan Lan.

## References

[1] He X Hong W Pan X Lu G Wei X. SARS-CoV-2 omicron variant: characteristics and prevention. MedComm (2020). 2021;2:838–45. 34957469 10.1002/mco2.110 PMC8693031

[2] Shi Y Wang G Cai XP. An overview of COVID-19. J Zhejiang Univ Sci B. 2020;21:343–60. 32425000 10.1631/jzus.B2000083 PMC7205601

[3] Li C Ren L. Recent progress on the diagnosis of 2019 novel coronavirus. Transbound Emerg Dis. 2020;67:1485–91. 32395897 10.1111/tbed.13620 PMC7272792

[4] Kim DH Kim D Moon JW Chae SW Rhyu IJ. Complications of nasopharyngeal swabs and safe procedures for COVID-19 testing based on anatomical knowledge. J Korean Med Sci. 2022;37:e88. 35315599 10.3346/jkms.2022.37.e88 PMC8938608

[5] Lee RA Herigon JC Benedetti A Pollock NR Denkinger CM. Performance of saliva, oropharyngeal swabs, and nasal swabs for SARS-CoV-2 molecular detection: a systematic review and meta-analysis. J Clin Microbiol. 2021;59:e02881–20. 33504593 10.1128/JCM.02881-20 PMC8091856

[6] Kanodia A Srigyan D Sikka K. Topical lignocaine anaesthesia for oropharyngeal sampling for COVID-19. Eur Arch Otorhinolaryngol. 2021;278:1669–73. 33001294 10.1007/s00405-020-06402-z PMC7528153

[7] Wilson NM Norton A Young FP Collins DW. Airborne transmission of severe acute respiratory syndrome coronavirus-2 to healthcare workers: a narrative review. Anaesthesia. 2020;75:1086–95. 32311771 10.1111/anae.15093 PMC7264768

[8] To KK Sridhar S Chiu KH. Lessons learned 1 year after SARS-CoV-2 emergence leading to COVID-19 pandemic. Emerg Microbes Infect. 2021;10:507–35. 33666147 10.1080/22221751.2021.1898291 PMC8006950

